# A Novel Glycoengineered Humanized Antibody Targeting DLK1 Exhibits Potent Anti-Tumor Activity in DLK1-Expressing Liver Cancer Cell Xenograft Models †

**DOI:** 10.3390/ijms252413627

**Published:** 2024-12-19

**Authors:** Koji Nakamura, Kota Takahashi, Izumi Sakaguchi, Takumi Satoh, Lingyi Zhang, Hiroyuki Yanai, Yukihito Tsukumo

**Affiliations:** Chiome Bioscience Inc., 3-12-1 Honmachi, Shibuya-ku, Tokyo 151-0071, Japan; ktakahashi@chiome.co.jp (K.T.); i.sakaguchi@renatherapeutics.com (I.S.); tsato@chiome.co.jp (T.S.); lingyi.zhang2@pfizer.com (L.Z.); hyanai@chiome.co.jp (H.Y.)

**Keywords:** Delta-like 1 homolog, CBA-1205, antibody-dependent cellular cytotoxicity, afucosylated antibody, hepatocellular carcinoma

## Abstract

Delta-like 1 homolog (DLK1), a non-canonical Notch ligand, is highly expressed in various malignant tumors, especially in hepatocellular carcinoma (HCC). CBA-1205 is an afucosylated humanized antibody against DLK1 with enhanced antibody-dependent cellular cytotoxicity (ADCC). The binding characteristics of CBA-1205 were analyzed by enzyme-linked immunosorbent assay and fluorescence-activated cell sorting assay. The ADCC activity of CBA-1205 was assessed. The anti-tumor efficacy of CBA-1205 was evaluated in xenograft mouse models, and toxicity and toxicokinetic profiles of CBA-1205 were evaluated in cynomolgus monkeys. CBA-1205 selectively bound to DLK1 among the Notch ligands and only to monkey and human DLK1. The binding epitope was between epidermal growth factor-like domains 1 and 2 of DLK1, which are not involved in any known physiological functions. The ADCC activity of CBA-1205 was confirmed using human peripheral blood mononuclear cells as effector cells. CBA-1205 as a single agent and in combination with lenvatinib demonstrated long-lasting anti-tumor efficacy, including tumor regression, in two liver cancer xenograft models. The toxicity and toxicokinetic profiles of CBA-1205 in cynomolgus monkeys were favorable. These findings suggest that CBA-1205 has the potential to be a useful therapeutic option for drug treatment in HCC. A phase 1 study is ongoing in patients with advanced cancers (jRCT2080225288, NCT06636435).

## 1. Introduction

Liver cancer is the sixth most common cancer worldwide, with an estimated 866,136 cases reported in 2022 [1]. Hepatocellular carcinoma (HCC) accounts for 90% of all liver cancers. Hepatitis B, hepatitis C, and non-alcoholic steatohepatitis are implicated in the development of HCC. The standard systemic treatments for advanced HCC are combination therapies with immune checkpoint inhibitors and vascular endothelial growth factor (VEGF) antibodies or monotherapies of tyrosine kinase inhibitors. However, treatment options are restricted for relapse/refractory patients after these treatments. Lenvatinib is a small-molecule multi-tyrosine kinase inhibitor against VEGFRs and fibroblast growth factor receptors (FGFRs) and one of the first-line and second-line agents for patients with unresectable advanced HCC who are ineligible for immune checkpoint inhibitors [2,3]. However, varying sensitivity to lenvatinib has been reported among patients with HCC [4], and several mechanisms of resistance to lenvatinib have been identified, such as enhanced epidermal growth factor receptor (EGFR) signaling and Wnt/β-catenin signaling [5]. Thus, the identification of novel therapeutic strategies for HCC is needed to improve the treatment of patients with HCC.

One of the causes of the unsatisfactory therapeutic response to standard treatments is the intratumor heterogeneity of HCC [6]. The presence of cancer stem cells, which possess the abilities for self-renewal and differentiation into multiple subtypes of tumor cells, is thought to be involved in promoting intratumor heterogeneity and drug resistance [7]. Therefore, cancer stem cells may be a target in the treatment of HCC.

Delta-like 1 homolog (DLK1) is a single transmembrane protein that is a member of the Notch ligand family. DLK1 consists of six epidermal growth factor (EGF)-like repeat sequences in the extracellular region, a disintegrin and metalloprotease (ADAM)-17 cleavage site in the juxtamembrane region, a transmembrane domain, and a short intracellular tail [8]. The extracellular region of DLK1 is cleaved by ADAM-17, and a fragment of the extracellular region is secreted into the blood as soluble DLK1. While the involvement of DLK1 in wound healing and inhibition of adipocyte differentiation has been reported, the physiological functions of DLK1 are largely unknown [9]. In the fetus, DLK1 is expressed in many tissues, but after birth, its expression is restricted to endocrine tissues (pituitary, adrenal, placenta, and pancreas) [8]. DLK1 expression has been reported in tissue stem/progenitor cells such as hepatoblasts, hepatic oval cells, mesenchymal stem cells, and prostate epithelial progenitor cells [10,11,12,13].

Several reports have linked DLK1 to cancer. Immunohistochemical analysis showed that DLK1 was expressed in HCC cells, but not in other liver diseases, such as hepatitis and cirrhosis [14]. DLK1 is one of the hepatic progenitor cell (HPC) markers and is co-expressed with other HPC markers (EpCAM, CK19, NCAM). HCC patients with higher levels of blood tumor markers (AFP, AFP-L3) have a higher expression of DLK1, along with simultaneous co-expression with other HPC markers. These patients also have a poor postoperative prognosis [15]. In a two-step hepatic carcinogenesis mouse model, the expression of DLK1 and cancer stem cell markers was found to be upregulated in liver tumors, and knockdown of DLK1 inhibited liver tumor growth [16,17]. DLK1 knockdown in HCC cell lines was shown to suppress colony formation in vitro and tumor growth in vivo [16]. In an experiment with 17 different liver cancer (mainly HCC) cell lines, the DLK1-positive sub-populations isolated from each cell line showed higher in vitro colony formation and cell proliferation activities and increased in vivo tumorigenic potential compared with DLK1-negative sub-populations [18]. DLK1-positive sub-populations have also been reported to express stem cell and progenitor cell markers (Nanog, Oct3/4, SOX2) and exhibit cancer stem cell–like characteristics, including resistance to chemotherapeutic agents [7,18]. Therefore, DLK1 may be a candidate cancer stem cell marker in HCC in addition to its function as a HPC marker.

CBA-1205 is a novel recombinant humanized IgG1/κ monoclonal antibody, discovered by Chiome Bioscience Inc., that specifically binds to human DLK1. The antibody-dependent cellular cytotoxicity (ADCC) of CBA-1205 was enhanced through the use of GlymaxX^®^ technology (ProBioGen AG, Berlin, Germany), which increases ADCC activity by the modification of the glycan structure of the Fc portion at Asn 297 [19,20]. A phase I, first in humans, study of CBA-1205 is ongoing in patients with advanced or recurrent solid tumors (jRCT2080225288, NCT06636435).

Here, we report the preclinical characterization of CBA-1205, including its efficacy in HCC in vitro and in vivo models [20] and safety and toxicokinetic profiles in non-human primates. We also demonstrate that CBA-1205 showed synergistic and durable efficacy in combination with lenvatinib in HCC pre-clinical animal models [21].

## 2. Results

### 2.1. Binding Affinity of CBA-1205 to DLK1

**Figure 1:**
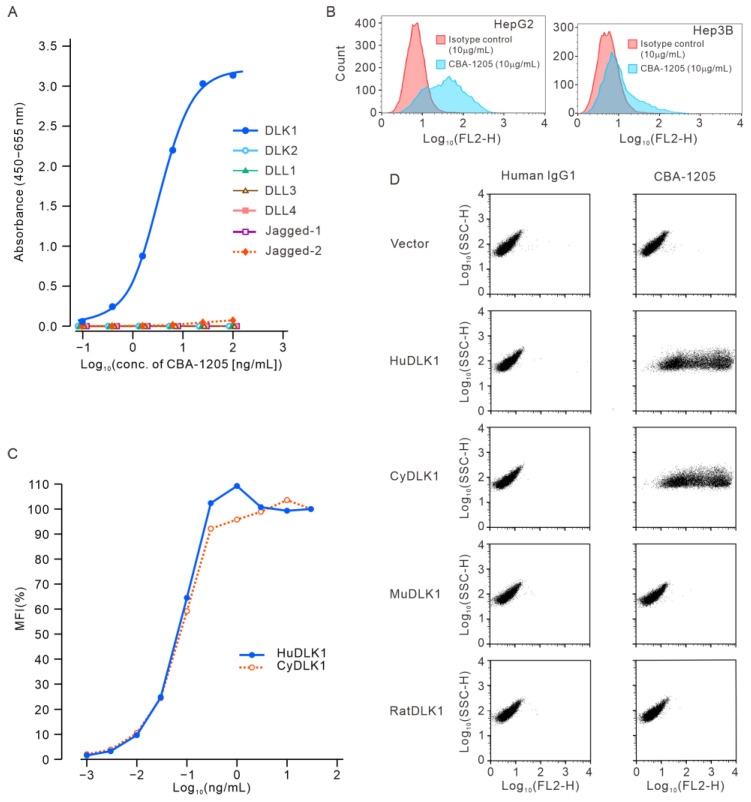
Binding characteristics of CBA-1205 to human DLK1. (**A**) ELISA of CBA-1205 binding to Notch ligand family proteins; (**B**) Flow cytometric assay of CBA-1205 binding to liver cancer cell lines that express endogenous DLK1; (**C**) Equivalent binding affinity of CBA-1205 to human and cynomolgus monkey DLK1 (HuDLK1 and CyDLK1, respectively); (**D**) Species specificity of CBA-1205 binding to human DLK1, cynomolgus monkey DLK1, mouse DLK1 (MuDLK1), and rat DLK1 (RatDLK1). The expression vectors (pcDNA3) encoding human, mouse, rat, and cynomolgus monkey DLK1, with a FLAG tag at the C-terminus, were transiently expressed into Expi293F™ cells. Reactivity of CBA-1205 (30 µg/mL) to cells transiently expressing the indicated DLK1 genes was analyzed by flow cytometry. MFI, mean fluorescence intensity; SSC-H, side scatter height; FL2, second fluorescence channel (for detection of phycoerythrin-labeled secondary antibody).

The Notch ligand family includes JAGGED-1, JAGGED-2, Delta-like protein (Dll)-1, Dll-3, Dll-4, DLK1, and DLK2 [8]. The binding activity of CBA-1205 to DLK1 and the other Notch ligand family proteins was evaluated by enzyme-linked immunosorbent assay (ELISA). The results showed that CBA-1205 bound only to DLK1, with a half maximal effective concentration (EC_50_) of 3.433 ng/mL [95% CI, 3.292–3.579] (Figure 1A).

We next evaluated the binding of CBA-1205 to Hep3B and HepG2 cells, human liver cancer cell lines that express endogenous DLK1 on the cell surface [16]. Flow cytometry analysis showed that CBA-1205 bound to both Hep3B and HepG2 cell lines (Figure 1B).

Flow cytometry analysis showed comparable binding affinity of CBA-1205 to human DLK1 and cynomolgus monkey DLK1, which were expressed in EXpi293F™ cells derived from the human embryonic kidney 293F cell line. The EC_50_ values were 0.070 µg/mL for human DLK1 and 0.076 µg/mL for cynomolgus monkey DLK1 (Figure 1C). The reactivity of CBA-1205 to human, monkey, rat, and mouse DLK1 was further evaluated by flow cytometry of Expi293F™ cells transiently transfected with a mammalian expression vector (pcDNA3) encoding the indicated full-length DLK1 constructs. Expression of all DLK1 constructs was confirmed by western blot analysis using anti-FLAG antibody. CBA-1205 bound to human and monkey DLK1, but did not bind to rat and mouse DLK1 (Figure 1D).

**Figure 2:**
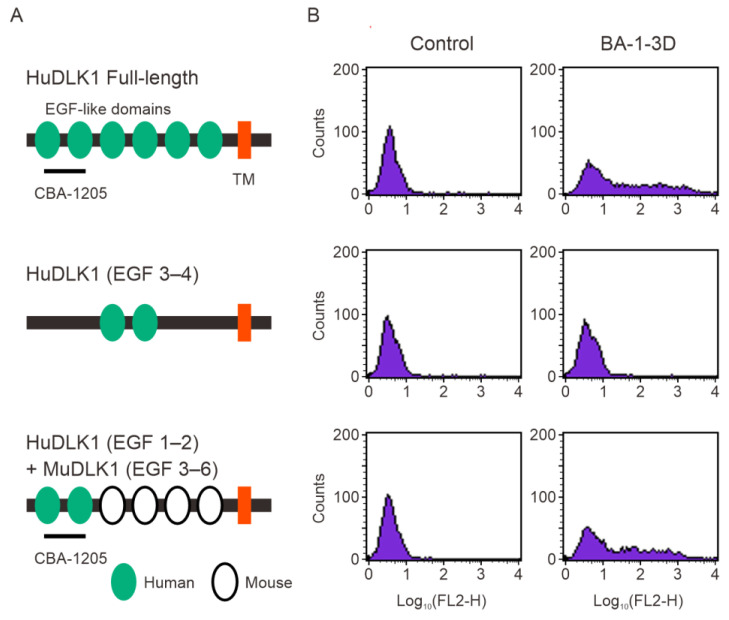
Epitope analysis of CBA-1205 antibody by flow cytometry. (**A**) Constructs of DLK1; (**B**) Flow cytometric assay of CBA-1205 binding to cells expressing various DLK1 constructs. HuDLK1, human DLK1; EGF, epidermal growth factor; EGF 3–4, EGF-like repeat 3–4 (hereafter abbreviated in the same manner); MuDLK1, mouse DLK1; BA-1-3-D, mouse anti-HuDLK1 antibody, based on which CBA-1205 was humanized and afucosylated.

DLK1 contains six EGF-like repeats in its extracellular region [8]. Epitope analysis using truncated DLK1 constructs and a chimeric construct of human and mouse DLK1 indicated that the DLK1 binding site of CBA-1205 is the EGF-like domains 1–2 (Figure 2).

### 2.2. CBA-1205 Has No Effect on Liver Cancer Cell Proliferation

**Figure 3:**
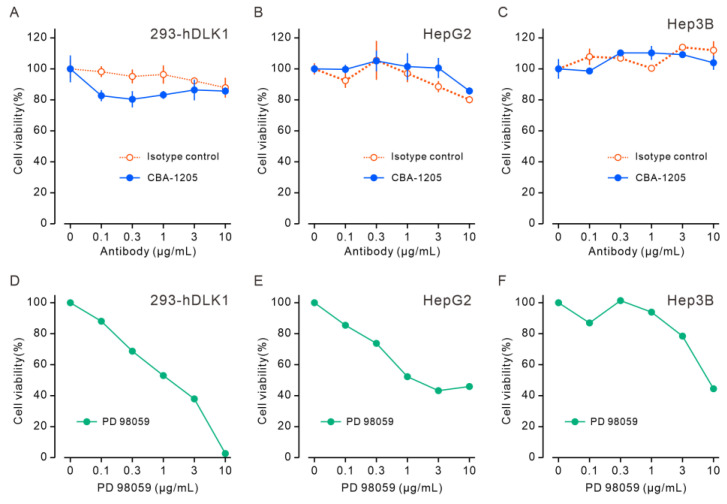
CBA-1205 does not affect cell proliferation of human liver cancer cell lines in vitro. (**A**–**C**) Cells were incubated with various concentrations of CBA-1205 at 37 °C in 5% CO_2_ for 96 h. Assays were performed in triplicate. Data are expressed as means ± SD. (**D**–**F**) Cells were incubated with various concentrations of the MEK inhibitor PD98059 at 37 °C in 5% CO_2_ for 96 h. (**A**,**D**) 293-hDLK1 cells stably expressing human DLK1; (**B**,**E**) HepG2 cells; (**C**,**F**): Hep3B cells.

DLK1 has been reported to promote cell proliferation [10,11,12,13]. DLK1 knockdown in liver cancer cell lines was shown to suppress colony formation in vitro and tumor growth in vivo [16]. The effect of CBA-1205 on the proliferation of 293-hDLK1 cells, which stably express human DLK1, and HepG2 and Hep3B cells was examined using Cell Counting Kit (CCK)-8 assays. A mitogen-activated protein kinase kinase (MEK) 1 inhibitor, PD98059, was used as the positive control for inhibition of cell proliferation. The results showed that CBA-1205 had no effect on the cell proliferation of HepG2 and Hep3B cells (Figure 3). These results indicate that CBA-1205 does not affect liver cancer cell proliferation.

### 2.3. ADCC Activity of CBA-1205

**Figure 4:**
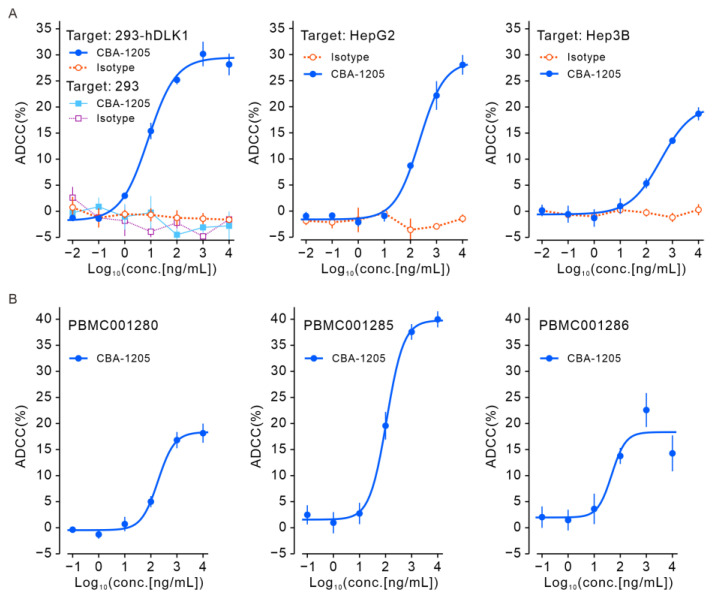
ADCC activity of CBA-1205 in vitro. (**A**) ADCC assays with the indicated cell lines as target cells and FcγRIIIA-expressing NK cells as effector cells; the E:T ratio was 40:1. Each point is mean ± SD (*n* = 3). (**B**) ADCC assays with HepG2 and three different lots of human peripheral blood mononuclear cells (PBMCs) as effector cells; the E:T ratio was 20:1. Each point is mean ± SD (*n* = 3). 293-hDLK1, a stable cell line established by transfection of 293 cells with a human DLK1 expression vector; ADCC, antibody-dependent cell-mediated cytotoxicity.

We next evaluated the ADCC activity of CBA-1205, which has enhanced ADCC activity by GlymaxX^®^ technology, against 293-hDLK1, Hep3B, and HepG2 cells that express DLK1. The ADCC activity of CBA-1205 was confirmed using the human Natural killer (NK) cell line TK176V, which expresses human FcγRIIIA, as effector cells (Figure 4A). We also observed ADCC activity using three lots of peripheral blood mononuclear cells (PBMCs) as effector cells (Figure 4B).

### 2.4. Antitumor Effects of Single-Agent CBA-1205 and Lenvatinib in Xenograft Mouse Models

**Figure 5:**
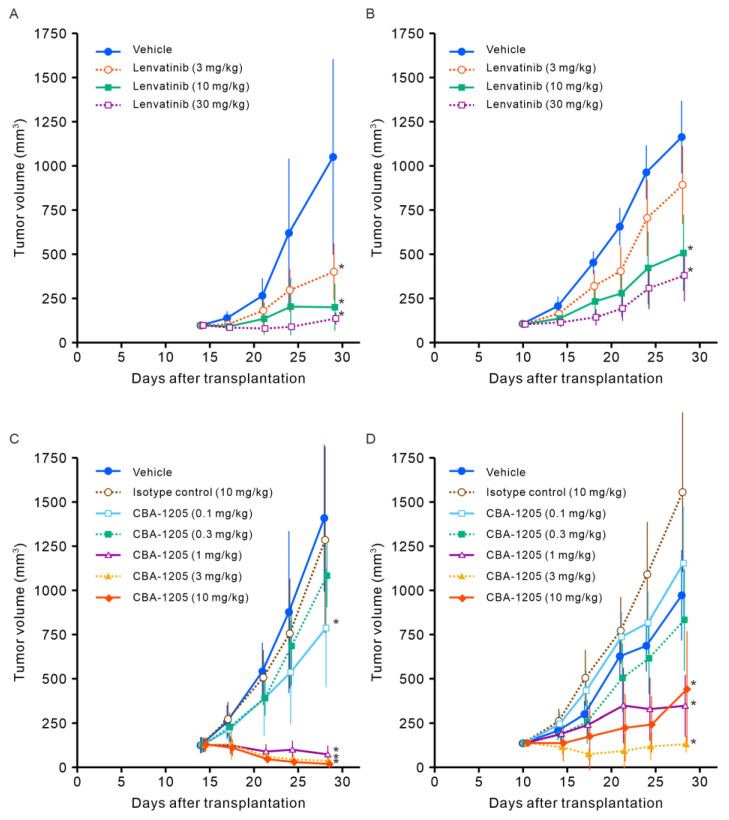
Anti-tumor efficacy of CBA-1205 and lenvatinib in two different liver cancer cell xenograft mouse models. Human hepatocellular carcinoma cell lines (Hep3B, HepG2) were subcutaneously transplanted in the right flank of NOD-SCID mice. When the tumor volume reached >100 mm^3^, mice were randomized to treatment groups (eight animals in each group). Lenvatinib was orally administered 5 days a week. CBA-1205 was intraperitoneally administered approximately every 3 days. (**A**) Hep3B model groups received vehicle (purified water) or lenvatinib alone; (**B**) HepG2 model groups received vehicle or lenvatinib alone; (**C**) Hep3B model groups received vehicle, isotype control IgG1/κ, or CBA-1205 alone on days 14, 17, 21, and 24; (**D**) HepG2 model groups received vehicle, isotype control IgG1/κ, or CBA-1205 alone on days 10, 14, 17, 21, and 24. Data are shown as means ± SD. Error bars indicate ± SD. * *p* < 0.05, by the two-tailed Dunnett’s multiple comparison test using the vehicle control group as the reference group.

The antitumor effects of CBA-1205 and lenvatinib, a standard treatment for HCC, were evaluated in Hep3B and HepG2 xenograft models using NOD/ShiJic-scidJcl (NOD-SCID) mice due to the higher engraftment rate for Hep3B and HepG2 xenografts in NOD-SCID mice than in other immunodeficient mice. Lenvatinib caused significant growth inhibition of Hep3B cell–derived tumors at 3 mg/kg to 30 mg/kg in a dose-dependent manner (*p* < 0.05 vs. vehicle control group by Dunnett’s test) (Figure 5A). The tumor volumes (means ± SD) on Day 29 in the control and 30 mg/kg lenvatinib groups were 1050.3 ± 553.8 and 136.2 ± 33.8 mm^3^, respectively. Lenvatinib also showed dose-dependent inhibition of growth of HepG2 cell–derived tumors (*p* < 0.05 vs. vehicle control by Dunnett’s test), but it only partially reduced tumor growth (Figure 5B). No weight loss was observed in mice administered lenvatinib at doses of 3 to 30 mg/kg.

CBA-1205 also demonstrated antitumor effects, including tumor regression, at doses of 1 mg/kg or higher in the Hep3B xenograft mouse model. The tumor volumes (means ± SD) on Day 28 in the vehicle, 10 mg/kg isotype control, and 1 mg/kg, 3 mg/kg, and 10 mg/kg CBA-1205 groups were 1408.8 ± 416.6, 1285.1 ± 527.6, 73.3 ± 50.0, 36.4 ± 34.2, and 18.6 ± 23.5 mm^3^, respectively. There were significant differences between the vehicle and CB-1205 (≥1 mg/kg) administered groups (*p* < 0.05 by the two-tailed Dunnett’s test) (Figure 5C). CBA-1205 also showed inhibitory effects on HepG2 cell–derived tumor growth, with maximum effects at 3 mg/kg (Figure 5D). The tumor volumes (means ± SD) on Day 28 in the vehicle, 10 mg/kg isotype control, and 1 mg/kg, 3 mg/kg, and 10 mg/kg CBA-1205 groups were 971.9 ± 256.4, 1555.5 ± 451.9, 346.9 ± 174.4, 130.5 ± 47.0, and 440.6 ± 328.6 mm^3^, respectively, with significant differences between vehicle and CB-1205 administered groups (*p* < 0.05 by the two-tailed Dunnett’s test). No weight loss was observed in mice administered CBA-1205 at doses of 0.1 to 10 mg/kg.

### 2.5. Lenvatinib in Combination with CBA-1205 in Xenograft Mouse Models

**Figure 6:**
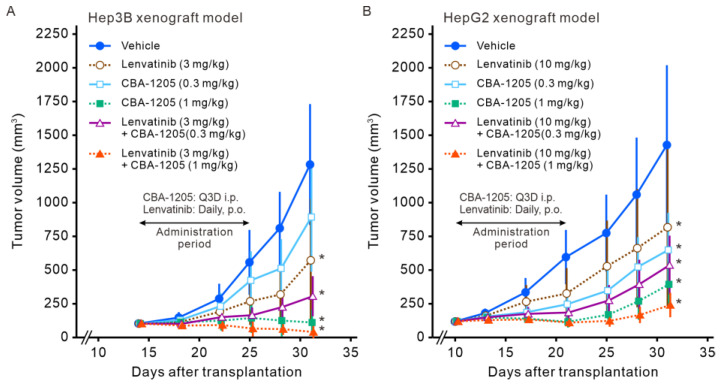
Combined effect of CBA-1205 and lenvatinib in liver cancer cell xenograft mouse models. (**A**,**B**) Human hepatocellular carcinoma cell lines (Hep3B, HepG2) were subcutaneously transplanted into the right flank of NOD-SCID mice. When the tumor volume reached >100 mm^3^, mice were randomized into dosing groups. CBA-1205 (0.3 and 1 mg/kg, twice a week, a total of four times) was administered intraperitoneally in combination with or without 3 mg/kg (for the Hep3B model) or 10 mg/kg (for the HepG2 model) of lenvatinib (daily, a total of 10 times) in mice (eight animals in each group). Vehicle was purified water for lenvatinib and phosphate-buffered saline for CBA-1205. Data are shown as means ± standard deviation (SD). Error bars indicate ± SD. * *p* < 0.05 by the two-tailed Dunnett’s multiple comparison test using the vehicle control group as the reference group.

We next examined the efficacy of CBA-1205 in combination with lenvatinib in the Hep3B and HepG2 xenograft models. In Hep3B xenograft mice, enhanced and long-lasting anti-tumor efficacy was observed in response to combination treatment compared with the findings with CBA-1205 treatment alone or lenvatinib treatment alone (Figure 6A); namely, significant differences were observed in tumor volumes on day 31 after transplantation between groups of mice receiving lenvatinb at 3 mg/kg with and without CBA-1205 at 1 mg/kg, and CBA-1205 at 0.3 mg/kg with and without lenvatinib at 3 mg/kg in a Tukey’s multiple paired comparison test as a post hoc test (*p* < 0.05 for each). Tumor growth of all mice treated with CBA-1205 and lenvatinib showed almost complete inhibition, even at 2 weeks after the last dosing. Notably, four out of eight mice treated with 1 mg/kg of CBA-1205 in combination with 3 mg/kg of lenvatinib showed tumor disappearance. The tumor growth was further followed up for an additional 7 days (by day 38) (Figure S1). All mice showed regrowth of their tumors in both groups treated with lenvatinib alone at 3 mg/kg or CBA-1205 alone at 1 mg/kg. In contrast, the tumors in four out of eight mice in the combination treatment group were still undetectable at day 38.

Enhanced inhibition of tumor growth was also observed with CBA-1205 at 1 mg/kg in combination with lenvatinib at 10 mg/kg in the HepG2 xenograft model (Figure 6B). This result was confirmed by a significant difference in tumor volumes on day 31 between these two groups by a Tukey’s multiple paired comparison test (*p* < 0.05).

### 2.6. Toxicity Study of CBA-1205 in Cynomolgus Monkey

CBA-1205 bound to human and monkey DLK1 but did not bind to rat and mouse DLK1 (Figure 1D). Therefore, the cynomolgus monkey was selected as the species for toxicology assessment of CBA-1205. In the 4-week intermittent intravenous dose toxicity study of CBA-1205 in cynomolgus monkeys, no adverse events leading to death or discontinuation of CBA-1205 occurred at doses of up to 100 mg/kg (the highest dose). In histopathological examination, very slight changes in liver were observed; namely, Kupffer cell hypertrophy and periportal/sinusoidal mixed inflammatory cell infiltration were observed in the 30 and 100 mg/kg groups of males and in the 100 mg/kg group of females. Based on the results of histopathological examination, we considered the no-observed-adverse-effect level of CBA-1205 to be 10 mg/kg for males and 30 mg/kg for females, when it was administered once weekly for 4 weeks intravenously. In the toxicokinetics analysis, the C_max_ and AUC_0–168h_ values on Day 1 and Day 22 increased in a dose-dependent manner (Table 1). No sex differences were noted for any parameter. Anti-CBA-1205 antibodies were not detected at any measurement point in any animal.

## 3. Discussion

In this study, we demonstrated that CBA-1205 showed specific binding to DLK1 and did not bind to any other Notch ligand family members. This suggests that CBA-1205 does not affect the various physiological functions mediated by the other ligands. DLK1 expression is restricted to specific tissues and organs, such as the hypothalamus-pituitary-adrenal axis and pancreas [22,23,24]. The specific binding of CBA-1205 to DLK1 and restricted expression of DLK1 in adults suggest potential advantages in terms of reduced or limited adverse effects of CBA-1205.

While DLK1 has been reported to promote cell proliferation [10,11,12,13], CBA-1205 had no effect on the cell proliferation of DLK1-expressing cells such as HepG2 and Hep3B cells (Figure 3). Most of the physiological functions of DLK1 identified thus far are explained by its interaction with Notch1. Previous studies showed that the EGF-like repeat 5–6 of DLK1 is involved in the interaction between DLK1 and Notch1, and the EGF-like repeat 4–5 of DLK1 is required for the interaction of DLK1 to DLK1 [9,25,26,27]. Another study showed that the juxtamembrane region after the EGF-like repeat 6 of DLK1 interacts with the C-terminal region of fibronectin to suppress Notch-independent differentiation into adipocytes [28]. Epitope analysis revealed that CBA-1205 binds to EGF-like repeat 1–2 of DLK1 (Figure 2). These results may suggest that CBA-1205 binds to DLK1 without affecting the physiological function of DLK1. As shown in Figure S2, sequence alignment of the EGF-like 1–2 domains for human DLK1, cynomolgus DLK1, mouse DLK1, rat DLK1, and human DLK2 show high similarity between human DLK1 and cynomolgus DLK1 but not between human DLK1 and mouse DLK1, rat DLK1, and human DLK2. These results support the finding that CBA-1205 binds to the EGF-like 1–2 domain of cynomolgus monkey DLK1, as well as that of human DLK1. There are no glycosylation sites within EGF-like domain 1–2 and no involvement of the carbohydrate chains of DLK1 in the binding and ADCC activity of CBA-1205.

Our results demonstrated that CBA-1205 showed antitumor activity against HepG2 and Hep3B xenograft models as a single agent and in combination with lenvatinib, regardless of lenvatinib sensitivity (Figure 5C,D and Figure 6). CBA-1205 does not directly inhibit cell proliferation, and complement-dependent cytotoxicity is unlikely to be involved in this antitumor activity, because the complement level is significantly reduced in this NOD-SCID line (NOD/ShiJic-scidJcl). The mechanism of the antitumor activity of CBA-1205 in vivo may be from ADCC activity against DLK1-expressing cells, as identified in the in vitro results (Figure 4 and Figure 5C,D). Since macrophages still remain in NOD/SCID mice, they may serve as effector cells for CBA-1205 and have high ADCC activity enhanced by afucosylation. In addition, antibody-dependent cellular phagocytosis by macrophages may be involved in these antitumor effects.

Immune checkpoint inhibitors, multi-tyrosine kinase inhibitors, and anti-VEGF antibodies are used to treat patients with HCC. However, some patients experience resistance to these agents. The intratumor heterogeneity of HCC contributes to cancer cell proliferation, metastasis, drug resistance, and recurrence, making treatment difficult and leading to poor prognosis [6]. The presence of tumor stem cells is thought to be one of the causative factors for intratumor heterogeneity and treatment resistance [6,7,29]. Previous studies showed that DLK1-expressing sub-populations in human liver cancer cell lines exhibit cancer stem cell–like properties, such as high proliferative capacity, high invasiveness, and resistance to chemotherapy [18]. HCC patients harboring DLK1-expressing tumors show a poor prognosis [15]. Therefore, depletion of DLK1-expressing cancer cells by CBA-1205 may be a useful HCC treatment strategy. Our results demonstrate the antitumor activity of CBA-1205 in the liver cancer models, suggesting a potential therapeutic strategy by depletion of DLK1-positive cancer stem cells in HCC by ADCC activity of CBA-1205.

Lenvatinib is the standard of care for HCC for patients who are not suitable for immune checkpoint inhibitors [2,3]. In phase 3 clinical trials of HCC, lenvatinib did not show a clear combination effect when combined with anti programmed cell death (PD)-1 antibodies; the combination of lenvatinib and anti-PD-1 antibody (pembrolizumab) did not demonstrate significant efficacy over lenvatinib plus placebo [30]. For this reason, lenvatinib has been used as a single agent in real-world clinical practice. Our results suggest that CBA-1205 has the potential for clinical use in combination with lenvatinib (Figure 6A,B).

Various mechanisms of lenvatinib resistance in HCC have been reported [5]. Activation of the β-catenin pathway by Frizzled-10 and Interferon Regulatory Factor (IRF)2 was revealed as one of the mechanisms of cancer stem cell expansion in the liver and resistance to lenvatinib [31]. DLK1 is expressed in cancer stem cells, and its expression is regulated by Wnt/β-catenin signaling [32,33,34]. Therefore, some HCC cases with resistance to lenvatinib may show increased expression of DLK1. Cancers with activated Wnt/β-catenin signaling are known as immuno-excluded tumors and are less likely to respond to immune checkpoint inhibitors [35]. CBA-1205 may be a potential therapeutic agent targeting DLK1 in immuno-excluded cancers.

Approximately 4–57% of HCC cases have been reported to express DLK1 [36]. Moreover, DLK1 expression is not uniform within tumor tissue, indicating intratumoral heterogeneity [37]. Therefore, it is important to stratify patients who may benefit from CBA-1205. DLK1-expressing HCCs have been reported to show activation of RAS, Yes-associated protein (YAP), or Notch intracellular domain pathways, in addition to activation of the Myc pathway [37,38,39]. In contrast, DLK1 was not expressed in HCCs with AKT pathway activation. Moreover, an increase in serum soluble DLK1 levels in HCC patients was found to correlate with tumor size [40]. Using these characteristics as patient stratification markers, it may be possible to efficiently identify patients who may benefit from CBA-1205.

DLK1 has also been found to be expressed in lung cancer, pancreatic cancer, ovarian cancer, endocrine cancer, and pediatric cancer [36]. An association between DLK1 expression and patient prognosis was reported in small cell lung cancer, non-small cell lung cancer [41], ovarian cancer [42], and gastrointestinal stromal tumor [43]. DLK1-expressing tumor cells have a higher proliferation rate, higher invasive capacity with epithelial mesenchymal transition, and resistance to treatment compared with cancer cells not expressing DLK1 [44]. Therefore, CBA-1205 has potential as a treatment option for cancers in addition to HCC that express DLK1.

The toxicity and toxicokinetic profiles of CBA-1205 identified in this study were favorable. DLK1 was reported to be expressed in the hypothalamus-pituitary-adrenal axis and pancreas [22,23,24]. The toxicity study of CBA-1205 in monkeys revealed no abnormal findings in DLK1-expressing tissues. While sufficient data to explain this safety finding have not been obtained, it appears to be related to the affinity of CBA-1205 for DLK1 or the amount of DLK1 expressed in these normal tissues.

## 4. Materials and Methods

### 4.1. Generation of CBA-1205

Recombinant protein corresponding to the extracellular region of human DLK1 (amino acids 26–244, accession No. NP_003827, GenBank) was mixed with the immunization adjuvant TiterMax Gold (Funakoshi Corp., Tokyo, Japan) and used to immunize female BALB/c mice. Lymphocytes from immunized mice and a mouse myeloma cell line (P3-X63-Ag8.653) were fused to establish anti-HuDLK1 monoclonal antibody–producing hybridomas. Anti-HuDLK1 antibody (clone BA-1-3D) was isolated as the parental antibody.

Humanized BA-1-3D (HuBA-1-3D) was generated by CDR grafting. The afucosylated version of HuBA-1-3D (product code name: CBA-1205) produced by GlymaxX^®^ technology was manufactured by ProBioGen AG (Berlin, Germany).

### 4.2. Enzyme-Linked Immunosorbent Assay (ELISA)

ELISA was performed using recombinant protein–coated 96-well plates. The following recombinant proteins were used at concentrations of 2.5–10 µg/mL: human DLK-1 (ab151926, Abcam, Cambridge, UK), human DLK2:Fc (AG-40A-0158-C010, AdipoGen, San Diego, CA, USA), human Jagged-1:Fc (AG-40A-0081-C010, AdipoGen), human Jagged-2:Fc (AG-40A-0155Y-C010, AdipoGen), human DLL1:Fc (AG-40A-0116Y-C010, AdipoGen), human DLL3 (His tag) (ab255797, Abcam), and human DLL4 (His-tag) (DLL4-2235H, Creative BioMart, Shirley, NY, USA). The coated plates were blocked with 1% BSA and 0.05% Tween 20 in phosphate-buffered saline (PBS) (blocking buffer) for 1 h at room temperature. CBA-1205 was added at various concentrations in 0.5% BSA and 0.025% Tween 20 in PBS (ELISA buffer), and the plates were incubated for 1 h at room temperature. After extensive washes with 0.05% Tween 20 containing PBS, the plates were incubated with Mouse Anti-Human IgG Kappa-HRP (1:4000) (SouthernBiotech, Birmingham, AL, USA) for 1 h at room temperature, washed, and incubated with TMB reagent (Nacalai Tesque, Kyoto, Japan) for 10 min before quenching with 50 µL of 1 mol/L H_2_SO_4_. The absorbance at 450 nm (655 nm as the reference) was measured with a microplate reader (iMark, Bio-Rad, Hercules, CA, USA).

### 4.3. Cell Culture

The 293 cell line (JCRB9068, Japanese Collection of Research Bioresources Cell Bank (JCRB), Osaka, Japan) was cultured in Dulbecco’s Modified Eagle’s Medium (DMEM, Thermo Fisher Scientific K.K, Tokyo, Japan). We generated 293-hDLK1 cells by stably transfecting a human DLK1 expression vector [14] into 293 cells; these cells were cultured in DMEM. HepG2 human liver cancer cells (JCRB1054, JCRB) were cultured in DMEM. Hep3B human liver cancer cells (86062703, European Collection of Authenticated Cell Cultures (ECACC)) were cultured in Eagle’s minimal essential medium (EMEM, Thermo Fisher Scientific K.K.). The culture media were supplemented with 10% (vol/vol) heat-inactivated fetal bovine serum (FBS, Hyclone, Logan, UT, USA), 50 U/mL penicillin, 50 mg/mL streptomycin, and 4 mM L-glutamine. All cell lines were cultured in a humidified atmosphere containing 5% CO_2_ at 37 °C.

### 4.4. Flow Cytometry

Cell suspensions at 0.2–1.0 × 10^6^ cells/100 µL were incubated with CBA-1205 at 4 °C. After two washes, the cells were incubated with phycoerythrin (PE)-labeled goat anti-human IgG (CAT:2040-09, SouthernBiotech, Birmingham, AL, USA) or PE-labeled goat F(ab’)2 anti-human Ig (CAT:2012-09, SouthernBiotech, Birmingham, AL, USA) at 4 °C. After two washes, the reactivity of CBA-1205 to DLK1 on the cell surface was analyzed using a flow cytometer (FACS Calibur, BD Biosciences, San Jose, CA, USA).

### 4.5. Cell Proliferation Assay

Cell proliferation of 293-hDLK1, HepG2, and Hep3B cells was evaluated using a Cell Counting Kit-8 (CCK-8) (DOJINDO, Kumamoto, Japan). Cells cultured in a 10 cm cell culture dish at 80% confluency were changed from 10% FBS–containing culture medium to serum-free medium and cultured overnight. The cells were then trypsinized, and cell suspensions were prepared with FBS-containing culture medium. Cells (2 × 10^4^ cells/50 µL) were plated in 96-well plates, and CBA-1205 or human IgG1/κ isotype control (Bio X Cell, Lebanon, NH, USA, 659518A1) was added at various concentrations (0, 0.1, 0.3, 1, 3, and 10 µg/mL in 100 µL volume), with triplicate wells for each concentration. The positive control PD98059 was added to wells (0, 0.3, 1, 3, 10, 30, and 100 µM) in triplicate. The cells were cultured for 96 h in 5% CO_2_ at 37 °C. WST-8 solution from the CCK-8 kit was added, and cells were incubated for 1–3 h in 5% CO_2_ at 37 °C. The absorbance at 450 nm was measured using a plate reader (iMark, BioRad, Hercules, CA, USA).

### 4.6. ADCC Assay

ADCC assays were performed at Chemicals Evaluation and Research Institute, Japan (CERI, Tokyo, Japan). The 293 (negative control), 293-hDLK1 (positive control), and liver cancer cell lines (HepG2 and Hep3B) were used as target cells. Human FcγRIIIA–expressing NK cells, TK176V (CERI), were cultured in Roswell Park Memorial Institute 1640 Medium (RPMI 1640, Thermo Fisher Scientific K.K.) containing 10% FBS, 10 ng/mL IL-2, and 1.25 µg/mL puromycin. The target cells were labeled with chromium-51 (^51^Cr, PerkinElmer, Inc., Waltham, MA, USA) for 1 h at 37 °C. The labeling efficiency was pre-optimized as 50 mCi per 1 × 10^6^ cells. The labeled target cells were plated in 96-well U-bottom plates (BD Falcon, Franklin Lakes, NJ, USA) at 5 × 10^3^ cells/50 µL/well. Various concentrations of CBA-1205 or human IgG1/k isotype control were added at 100 µL/well, and the cells were cultured for 1 h at 37 °C. Next, 2 × 10^5^ cells (50 µL) of TK176V cells as effector cells (effector:target = 40:1) were added to the wells, and the plates were cultured for 4 h in 5% CO_2_ at 37 °C. The amount of ^51^Cr release (CPM) in 50 µL culture medium was measured by a gamma counter (2480 WIZARD2 auto gamma counter, PerkinElmer, Inc.).

Human peripheral blood mononuclear cells (hPBMCs) were also used as effector cells. Three different lots of PBMCs (PBM001280, PBM001285, PBM001286) (BIOPREDIC International, Saint-Grégoire, France) were used in the experiments. hPBMCs were cultured for one day in RPMI1640 medium containing 10% FBS, 10 ng/mL human IL-2, and 500 ng/mL human GM-CSF (FUJIFILM Wako Pure Chemical Corporation, Osaka, Japan). The effector/target ratio was 20:1.

ADCC activity (%) was calculated using the following formula:

cytotoxicity activity (%) = (each well CPM − spontaneous release CPM)/(max release CPM − spontaneous CPM) × 100

ADCC activity (%) = (cytotoxicity at antibody addition − cytotoxicity with no antibody)

### 4.7. Xenograft Experiments

Female NOD/ShiJic-scidJcl mice (NOD-SCID), 6–8 weeks old (Japan CLEA, Tokyo, Japan), were used in this study. Hep3B (1 × 10^7^ cells/mL) or HepG2 (5 × 10^7^ cells/mL) cells were prepared in a 1:1 mixture of PBS and BD Matrigel, reduced growth factor, phenol-red free (BD Pharmingen, San Jose, CA, USA). Cell suspensions (100 µL) were subcutaneously transplanted into the right flank of NOD-SCID mice. When the tumor volume reached > 100 mm^3^, the mice were randomized to treatment groups. The vehicle (PBS), human IgG1/κ isotype control, and CBA-1205 were intraperitoneally administered to the mice at the indicated dosage (0.1, 0.3, 1, 3, and 10 mg/kg) twice a week (8 mice in each dosing group). The vehicle (purified water for lenvatinib) or lenvatinib mesylate (79A59K, Eisai, Tokyo, Japan) was orally administered once a day at the indicated dosage (3, 10, and 30 mg/kg) for the indicated periods (8 mice in each dosing group). In the combination dosing study, CBA-1205 (0.3 and 1 mg/kg, twice a week, a total of four times) was administered intraperitoneally with or without 10 mg/kg lenvatinib (daily, a total of 10 times) in Hep3B and HepG2 xenograft mice (8 animals in each group). Animals in the control group (*n* = 8) received the vehicle (purified water orally and PBS intraperitoneally) on the same schedule.

Tumor growth was measured with calipers in two perpendicular dimensions, and tumor volume (mm^3^) was calculated using the formula (width^2^ × length) × π/6. The body weight and the general symptoms of the animals were measured and observed at the time of administration throughout the experiment.

All animal experiments in this study were approved and conducted in accordance with the Institutional Animal Care and Use Committee guidelines of Chiome Bioscience Inc. (Tokyo, Japan).

### 4.8. Epitope Assays

Three DLK1 expression vectors were constructed for epitope analysis. The expression vector encoding full-length HuDLK1 was previously described [14]. The (EGF3–4) and human/mouse chimera DLK1 (human EGF 1–2 connected with mouse EGF3–6) constructs were generated by PCR. The sequences of PCR primers were as follows: forward primer: 5′-gcggccgcgcctgctcctcggccccc-3′, reverse primer: 5′-tctagagaggctgttggccacgatctcgc-3′ for HuDLK1 EGF 3-4; and forward primer, Y403-Not: 5′-gcggccggctgaatgcttcccggcc-3′, reverse primer, Y413: 5′-gtccacgcaagttccgttgttggcacaggg-3′, forward primer, Y444: 5′-ccctgtgccaacaacggaacttgcgtggac-3′, and reverse primer, Y441: 5′-tctagattagatctcctcatcacc-3′ for HuDLK1 (EGF1–2)/mouse DLK1 (EGF3-6) chimera. PCR products were verified by DNA sequencing, and the fragments were sub-cloned into the pME18SNeo plasmid, which carries the signal sequence of CD8, His tag, and transmembrane and cytoplasmic domains of FXYD5 [14]. The three expression vectors were transiently expressed into CHO-K1 cells using Lipofectamine 2000 reagent (Thermo Fisher Scientific K.K.). At 24 h after transfection, the cells were trypsinized and subjected to flow cytometry analysis for epitope analysis of BA-1-3D antibody.

### 4.9. Cross-Reactivity of CBA-1205 to Human, Cynomolgus Monkey, Mouse, and Rat DLK1

The expression vector encoding human DLK1 was constructed as described previously [14]. Mouse DLK1 cDNA was isolated as described previously [10]. The PCR amplified cDNA fragment of mouse DLK1 was cloned into the pcDNA3 vector (Invitrogen, Carlsbad, CA, USA). Rat DLK1 cDNA was synthesized using the amino acid sequence (Genbank accession No. AAI67752.1) with 5′-kozak sequences (GCCACC) and EcoRI restriction enzyme site (GAATTC) and 3′ HindIII site. The synthesized rat DLK1 cDNA was digested with EcoRI and HindIII and subcloned into the pcDNA3 vector. Cynomolgus monkey DLK1 cDNA was synthesized using amino acid sequences (Accession No. XP_015309693). The PCR-amplified cDNA of cynomolgus monkey DLK1 with a 3′-FLAG tag was subcloned into the pcDNA3 vector. The sequences of human, mouse, rat, and cynomolgus monkey DLK1 were verified by DNA sequencing.

To analyze the cross-reactivity of CBA-1205 to human, mouse, rat, and cynomolgus monkey DLK1, the expression vectors encoding human, mouse, rat, and cynomolgus monkey DLK1 were transiently expressed into Expi293F™ cells (Thermo Fisher Scientific K.K.) using the ExpiFectamine 293 Transfection Kit (Thermo Fisher Scientific K.K.) following the manufacturer’s instructions. After 48 h, the transfected cells were suspended with BAMBANKER (GC Lymphotec, Tokyo, Japan) and stored at −80 °C until use. Cross-reactivity of CBA-1205 was analyzed by flow cytometry as described above.

### 4.10. Toxicity Study in Cynomolgus Monkeys

The general toxicity including safety pharmacology, immunotoxicity, serum cytokine profile, toxicokinetics (at 1st and 4th doses), and antibody analysis of CBA-1205 was evaluated in cynomolgus monkeys under GLP compliance. CBA-1205 was intravenously administered once weekly for 4 weeks at 0 (vehicle: 25 mM histidine-HCl buffer (pH 6.0)), 10, 30 and 100 mg/kg (dose volume: 5 mL/kg) to four male and four female cynomolgus monkeys per each dose group. Two males and two females were added to the 0 and 100 mg/kg groups to assess the reversibility of toxicity during a 4-week recovery period.

### 4.11. Statistical Analysis

Concentration-response curves were analyzed using GraphPad Prism version 10.2.0 (GraphPad Software, La Jolla, CA, USA), fitting a 4-parameter logistic model. EC_50_ values and its 95% CI were estimated from the curves. Tumor volumes are expressed as means ± standard deviation (SD). Statistical analysis of tumor size differences on the final study day were evaluated by the two-tailed Dunnett’s test (vs. vehicle treatment group), and a Tukey’s multiple paired comparison test when the significant results were observed in the Dunnett’s test, using the statistical program MEPHAS (http://www.gen-info.osaka-u.ac.jp/MEPHAS/dunnett-e.html, accessed on 26 April 2024). *p* < 0.05 indicated a significant difference. Toxicokinetic parameters following intravenous bolus administration of CBA-1205 were analyzed using the methods of non-compartment analysis and linear trapezoidal linear interpolation in Phoenix™ WinNonlin version 6.4 (Certara LP, Princeton, NJ, USA).

## 5. Conclusions

We demonstrated the long-lasting anti-tumor efficacy of the afucosylated humanized anti-DLK1 antibody CBA-1205 as a single agent and in combination treatment with lenvatinib in two liver cancer cell xenograft mouse models. Additionally, the toxicity and toxicokinetics profiles of CBA-1205 in cynomolgus monkeys were favorable. These findings suggest that CBA-1205 has the potential to be a useful therapeutic option for patients with HCC.

DLK1 expression has been reported in various cancers in addition to HCC, and thus CBA-1205 may also be a therapeutic option for patients with other cancers. From these findings, we have decided to conduct a clinical study of CBA-1205, where the safety and preliminary efficacy of CBA-1205 is currently being evaluated in patients with advanced cancer, including HCC, in a phase I study (jRCT2080225288, NCT06636435).

## Figures and Tables

**Table 1:** Toxicokinetic parameters of CBA-1205 in cynomolgus monkeys.

			Day 1 Mean ± SD			Day 22 Mean ± SD		
Dose (mg/kg)	Sex	n	C_max_ (µg/mL)	t_1/2_ (h)	AUC_0–168 h_ (µg·h/mL)	C_max_ (µg/mL)	t_1/2_ (h)	AUC_0–168 h_ (µg·h/mL)
10	Male	4	235 ± 17	115 ± 8	19,200 ± 2500	387 ± 64	174 ± 29	39,900 ± 9000
	Female	4	222 ± 8	143 ± 29	17,400 ± 1700	360 ± 27	195 ± 59	37,200 ± 4800
30	Male	4	749 ± 125	109 ± 25	56,100 ± 7300	1320 ± 70	92.6 ± 13.0	106,000 ± 8000
	Female	4	702 ± 114	100 ± 10	56,200 ± 10,700	1330 ± 210	92.9 ± 17.7	112,000 ± 20,000
100	Male	6	2370 ± 430	72.9 ± 11.9	169,000 ± 28,000	4480 ± 740	88.3 ± 18.1	325,000 ± 58,000
	Female	6	2360 ± 370	82.9 ± 11.2	172,000 ± 15,000	4320 ± 280	81.6 ± 14.3	338,000 ± 38,000

## Data Availability

All data generated and analyzed during this study are included in this manuscript; further inquiries can be directed to the corresponding authors.

## Supplementary Materials

The following supporting information can be downloaded at: https://www.mdpi.com/article/10.3390/ijms252413627/s1.
