# Supplementary material for: A Novel Glycoengineered Humanized Antibody Targeting DLK1 Exhibits Potent Anti-Tumor Activity in DLK1-Expressing Liver Cancer Cell Xenograft Models
Source: Int J Mol Sci. 2024 Dec 19;25(24):13627. doi: 10.3390/ijms252413627 (PMC11679542; doi:10.3390/ijms252413627)
Supplement: Supplementary file 1 [file ijms-25-13627-s001.zip › ijms-3300976-supplementary.pdf]

Supplemental Material

A novel glycoengineered humanized antibody targeting DLK1 exhibits potent anti-tumor activity in DLK1-expressing liver cancer cell xenograft models

Koji Nakamura [1*], Kota Takahashi [1], Izumi Sakaguchi [1], Takumi Sato [1], Lingyi Zhang [1], Hiroyuki Yanai [1], Yukihito Tsukumo [1*]

[1]Chiome Bioscience Inc., 3-12-1 Hommachi Shibuya-ku, Tokyo 151-0071, Japan

*Correspondence: knakamura@chiome.co.jp; ytsukumo@chiome.co.jp

Table of contents

1. Supplemental Figure S1 p.2
2. Supplemental Figure S2 p.3

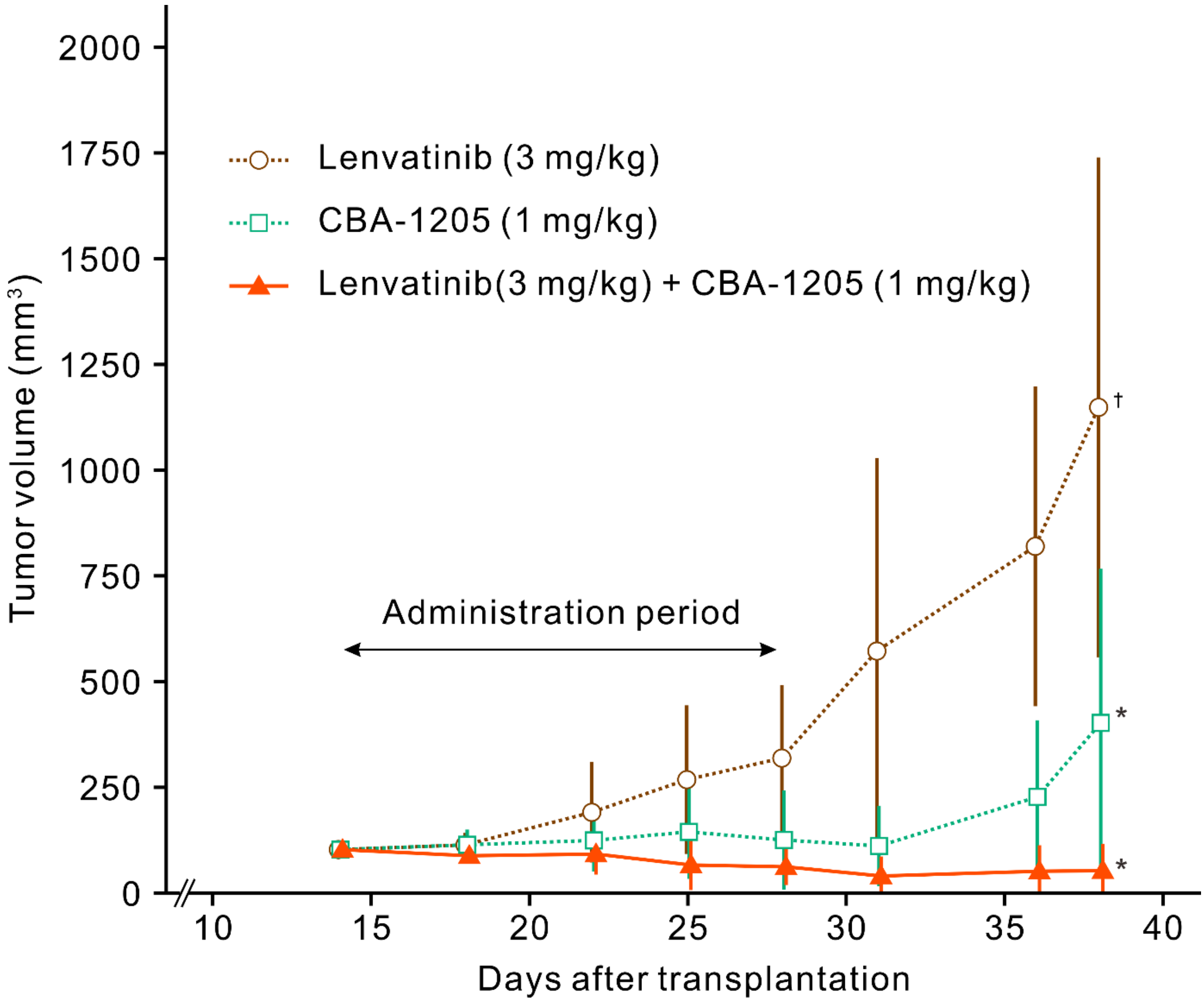

Figure S1. Combined effect of CBA-1205 (1 mg/kg) and lenvatinib (3 mg/kg) on tumor growth in a Hep3B human liver cancer cell xenograft model.

The growth of Hep3B tumor xenografts in NOD-SCID mice shown in Figure 6B was followed up until 38 days after transplantation in the 3 groups of mice (8 animals in each group), which were intraperitoneally administered 3 mg/kg of lenvatinib (daily, a total of 10 times), CBA-1205 (1 mg/kg, twice a week, a total of four times), and both during the period indicated in this figure, respectively. Data are shown as mean ± standard deviation (SD). Error bars indicate ± SD.

* $p < 0.05$ by the two-tailed Dunnett multiple comparison test using the lenvatinib treatment group as the reference group; † The mean and SD of the seven animals are shown for the lenvatinib alone-administered group only on day 38, because one animal was sacrificed on day 36 due to its tumor volume exceeding 1,500 mm^3.

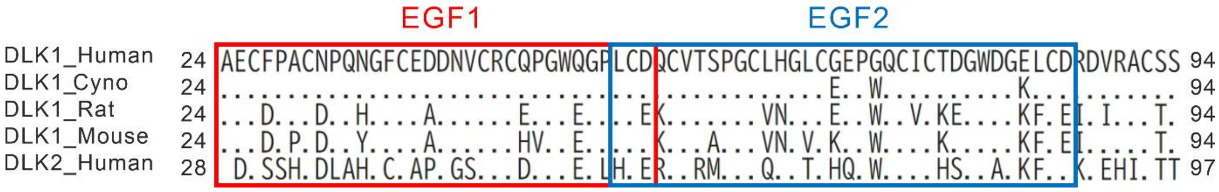

Figure S2. Sequence alignment of EGF-like domains 1-2 for human, cynomolgus, mouse, rat DLK1 and human DLK2

EGF-like domains 1-2 of Human DLK1 (Accession No. NP_003827), cynomolgus DLK1 (XP_015309693), mouse DLK1 (NP_034182), rat DLK1 (NP_446196), and human DLK2 (NP_076421) were aligned. The EGF-like domains 1 and 2 are boxed in red and blue, respectively. Identities are displayed as dots (.), with mismatches displayed as single letter abbreviations.
